# Phase Analysis and Thermoelectric Properties of Cu-Rich Tetrahedrite Prepared by Solvothermal Synthesis

**DOI:** 10.3390/ma15030849

**Published:** 2022-01-23

**Authors:** Karolina Zazakowny, Artur Kosonowski, Adrianna Lis, Oleksandr Cherniushok, Taras Parashchuk, Janusz Tobola, Krzysztof T. Wojciechowski

**Affiliations:** 1 Thermoelectric Research Laboratory, Faculty of Materials Science and Ceramics, AGH University of Science and Technology, 30-059 Krakow, Poland; karolina.zazakowny@agh.edu.pl (K.Z.); arturk@agh.edu.pl (A.K.); adlis@agh.edu.pl (A.L.); sashach@agh.edu.pl (O.C.); parashchuk@agh.edu.pl (T.P.); 2 Faculty of Physics and Applied Computer Science, AGH University of Science and Technology, 30-059 Krakow, Poland

**Keywords:** Cu-rich tetrahedrites, solvothermal synthesis, crystal structure, electronic structure, thermoelectric properties, liquid-like materials

## Abstract

Because of the large Seebeck coefficient, low thermal conductivity, and earth-abundant nature of components, tetrahedrites are promising thermoelectric materials. DFT calculations reveal that the additional copper atoms in Cu-rich Cu_14_Sb_4_S_13_ tetrahedrite can effectively engineer the chemical potential towards high thermoelectric performance. Here, the Cu-rich tetrahedrite phase was prepared using a novel approach, which is based on the solvothermal method and piperazine serving both as solvent and reagent. As only pure elements were used for the synthesis, the offered method allows us to avoid the typically observed inorganic salt contaminations in products. Prepared in such a way, Cu_14_Sb_4_S_13_ tetrahedrite materials possess a very high Seebeck coefficient (above 400 μVK^−1^) and low thermal conductivity (below 0.3 Wm^−1^K^−1^), yielding to an excellent dimensionless thermoelectric figure of merit *ZT* ≈ 0.65 at 723 K. The further enhancement of the thermoelectric performance is expected after attuning the carrier concentration to the optimal value for achieving the highest possible power factor in this system.

## 1. Introduction

Due to its unique property to interconvert heat and electricity, thermoelectric (TE) materials can be used for the construction of two main types of devices [1]. The first ones are thermoelectric generators (TEG), which directly convert waste heat into electric energy based on the Seebeck effect [2]. The other ones are Peltier’s cooling modules, so-called thermoelectric heat pumps, which can operate using the Peltier effect [3]. To create a temperature gradient, which is the necessary condition to induce the electro-motive force in TEG elements, the waste heat from the combustion gasses, nuclear reactors, or even human bodies can be used [4]. Thermoelectric generators have become especially important in the development of modern electronically powered devices where recovering waste energy is significant in terms of the price and eco-friendly nature [5,6].

The efficiency of thermoelectric material is defined by the dimensionless thermoelectric figure of merit ZT  =  S^2^σT/κ_el_+κ_L_, where S is the Seebeck coefficient, σ is the electrical conductivity, *T* is the temperature, and κ_el_, κ_lat_ are the electronic and lattice components of the thermal conductivity [7,8]. As the Seebeck coefficient, electrical conductivity, and electronic thermal conductivity are interrelated through the carrier concentration and particularities of the band structure, the development of the highly efficient TE materials requires specific properties (narrow bandgap, multivalley band structure, high mobility, high solubility of dopants, intrinsically low κ_lat_) [9,10,11]. Such an unusual combination of properties in one compound was found in the heavily-doped semiconductors, e.g., PbTe [12,13,14], Bi_2_Te_3_ [15,16], GeTe [17,18], CoSb_3_ [19,20], which are excellent thermoelectric materials. The limiting factors, which restrict their widespread utilization, are the costs of production, environmental friendliness, and efficiency of energy conversion. Therefore, many efforts of the thermoelectric community are now dedicated to the development of new classes of the materials, e.g., Mg_2_X (X = Si, Sn) [21], SnSe [22,23], argyrodites [24], and tetrahedrites [25,26,27], which better meet the modern industrial requirements.

In the last decades, more and more sulfide-based thermoelectric materials have been reported as excellent candidates for the construction of TEG converters [28]. Many transitional metal sulfides show good TE performance caused by their ultralow lattice thermal conductivity κ_L_ and promising electronic properties [29]. They also possess excellent features for widespread commercial use, such as non-toxicity and low price caused by high abundance [30]. Within the promising sulfide-based thermoelectrics, there are also materials that crystallize in the tetrahedrite-type structure [25,31,32]. The early investigation of the naturally-occurring Cu_12_Sb_4_S_13_ mineral has opened a large playground for this promising family of compounds [33,34]. Tetrahedrite minerals, which may have large potential for thermoelectric applications, can be extended to the compounds with the general formula A_10_^+^B_2_^2+^X_4_^3+^Y_13_^2-^, where A is Cu or Ag; B is Fe, Zn, Ni, Co, or Mn; X is Sb or As; and Y is for S [33,35].

The tetrahedrite materials are environmentally friendly and cheap in comparison to the well-established TE materials, which usually consist of expensive and toxic elements. Thermoelectric materials based on Cu_12_Sb_4_S_13_ possess very low lattice thermal conductivity (κ_L_ ≈ 0.5–0.9 Wm^−1^K^−1^) and promising values of the Seebeck coefficient (S ≈ 60–300 μVK^−1^) over the wide temperature range [36,37]. Yan et al. reported that tetrahedrite-based samples can possess extremely low lattice thermal conductivity (as low as ~0.25 Wm^−1^K^−1^ or even lower) [37], which makes them very promising candidates for further research.

Tetrahedrite-based materials are commonly synthesized using a solid-state reaction method. Subsequently, synthesized ingots should be annealed for a few weeks to obtain homogenous materials [25,38]. Alternatively, the TE tetrahedrites can be prepared using the naturally existing tetrahedrite minerals, which should be mixed with dopants and grounded in high-energy ball mills. In the next step, the obtained powders should be compacted and sintered [39]. However, due to high synthesis/sintering temperatures and long-term preparation processes, both described methods require enormous energy consumption. The alternative and low-energy-consuming methods for the preparation of tetrahedrite-based materials are based on solvothermal synthesis, e.g., in ethanol solution from inorganic chlorides and thiourea. CuCl_2_, SbCl_3_, and NH_2_CSNH_2_ should be mixed in absolute ethanol to form a heterogeneous mixture. In this case, the reaction occurs in an autoclave for 14–20 h at a remarkably low temperature of ~430 K [40]. However, the generation of the ultrahigh pressures inside an autoclave requires additional safety control, and the resulting products usually contain nonreacted precursors.

In this work, we offer a novel approach for the fabrication of TE materials, which is based on the solvothermal method. Particularly, to prepare Cu-rich Cu_14_Sb_4_S_13_ tetrahedrite, pure elemental powders were used as precursors, and ethanol, typically employed for solvothermal synthesis, was changed with the 1-(2-aminoethyl) piperazine, serving both as a solvent and reagent. The unique advantage of this method is that the synthesis can be carried out in an open vessel at relatively low temperatures and under atmospheric pressure. Moreover, the proposed method helps to avoid inorganic contaminations from substrate salts. The prepared Cu_14_Sb_4_S_13_ materials show a high thermoelectric dimensionless figure of merit *ZT* ≈ 0.65 at 723 K, which is mostly connected with the high Seebeck coefficient S, and low thermal conductivity κ. The measured thermal conductivity κ, for the Cu_14_Sb_4_S_13_ tetrahedrite, is equal to 0.17–0.32 Wm^−1^K^−1^, which is in the range of the lowest values of the thermal conductivity observed in the light-weight sulfides.

## 2. Materials and Methods

### 2.1. Synthesis

Synthesis of Cu-rich tetrahedrite was conducted by a single-step solvothermal reaction, where 1-(2-aminoethyl) piperazine (AEP) (Alpha Aesar, Haverhill, MA, USA, 98%) plays the role of the solvent as well as the reagent. AEP was placed in a double-neck round-bottom flask with added elemental powders of Cu (Alpha Aesar, Haverhill, MA, USA, 99.99%), Sb (Alpha Aesar, Haverhill, MA, USA, 99.99%), and S (Alpha Aesar, Haverhill, MA, USA, 99.999%). All syntheses were carried out in an oil bath at 200 °C. The syntheses were performed with continuous mixing using a magnetic stirrer with a heating plate. Samples described in this work were obtained in reactions carried out for one, two, five, and ten days, respectively. Obtained powders were filtered out using a Büchner funnel and washed at least three times with distilled water and acetone. After that, drying of powders was performed at 70 °C. The powders were subjected to structural analysis.

### 2.2. Sintering

The resultant powders were densified by pulsed electric current sintering (PECS) technique at 723 K for 20 min in 12.8 mm diameter graphite mold under axial compressive stress of 40 MPa in an argon atmosphere. The heating rate was 70 K/min, and the cooling rate was 20 K/min. Compacted pellets with a diameter of 12.8 mm and thickness of 2.5 mm were obtained and annealed in argon atmosphere for 5 h at 673 K for sample homogenization. Annealed pellets were polished and cut for transport property measurements.

### 2.3. Characterization

Powder X-ray diffraction (XRD) was performed on the BRUKER D8 Advance diffractometer (Bruker, Billerica, MA, USA) using Ni-filtered Cu-K_α_ radiation (λ = 1.5406 Å Δ2Ѳ = 0.007°, 2Ѳ range 10–90°). The multi-phase Rietveld refinement was carried out using GSAS II software (version 5149, 2010, Chicago, IL, USA) [41].

Microstructure investigation and chemical composition analyses were performed using scanning electron microscopy (SEM) and energy-dispersive X-ray spectroscopy (EDS) on the NOVA NANO SEM 200 (FEI EUROPE COMPANY, Eindhoven, Netherlands) microscope.

The Seebeck coefficient S and electrical conductivity σ were measured with NETZSCH SBA 458 Nemesis (Netzsch, Selb, Germany) in argon flow over the temperature range of 300–723 K. The electrical conductivity was measured using a four-probe method, applying the constant current of 1 mA through the sample. The Seebeck coefficient was estimated by creating temperature gradients with the help of the microheaters, which were placed below the two sample edges. The resulting voltage was measured by the thermocouples and then used for the calculation of the Seebeck coefficient. Thermal diffusivity α was measured by employing the NETZSCH LFA 457 equipment (Netzsch, Selb, Germany) using InSb high-temperature detector, and specific heat capacity C_p_ was estimated using the Dulong-Petit limit. The samples were first spray-coated with a thin layer of graphite to minimize errors from the emissivity of the material and laser beam reflection caused by the shiny pellet surface. Thermal conductivity was calculated using the equation κ = ρC_p_α, where ρ is the density determined by Archimedes’ principle at the discs obtained from PECS. Hall effect was investigated by applying the four-probe method in constant electric and magnetic fields (H = 0.9 T) and current through a sample of 100 mA. In order to measure sample grain sizes, analyzed powders were suspended in water and then sonicated for 3 min. Then, suspensions were analyzed with the dynamic light scattering method with a Zetasizer Nano-ZS, manufactured by Malvern Instruments (Malvern, PA, USA).

### 2.4. Electronic Structure Calculations

The electronic band structure calculations of densities of states (DOS) and dispersion E(**k**) curves of ordered Cu_12_Sb_4_S_12_ and Cu_12_Sb_4_S_13_ compounds were performed using the Korringa–Kohn–Rostoker (KKR) technique based on the Green function methodology [42]. In the case of disordered Cu_12+x_Sb_4_S_13_ alloys, the KKR was combined with the coherent potential approximation (CPA) [43,44], which explicitly treats the chemical disorder introduced by an excess of Cu in a random way. In these computations, the crystal potential was constructed within the local density approximation employing the exchange-correlation potential of Perdew and Wang [45]. The Fermi energy was determined precisely by the generalized Lloyd formula [46], which is especially important in systems lying on the verge of a metal-semiconductor limit. For all compositions, the experimental lattice parameters and atomic coordinates determined from Rietveld refinements were used. It is worth noting that in Cu_12+x_Sb_4_S_13_ alloys, Cu atoms occupied three crystallographic sites (12*d*, 12*e*, 24*g*), and the Cu excess was modeled by ‘alloying’ copper atoms (with nuclear charge Z = 29) and empty spheres (Z = 0), randomly occupying the 24*g* site, called Cu(3). However, in order to better understand the energetic distribution of copper atoms and the preference of the crystallographic position, we performed accurate total energy KKR-CPA calculations for several possible configurations of Cu atoms. In particular, we intended to check whether the excess Cu in this system is related to occupying only a new position Cu(3) or whether it may cause an incomplete filling of positions Cu(1) and Cu(2), as well. For well-converged crystal potential and atomic charges (below 1 meV and 10^−3^ e, respectively), the total-, site-, and l-decomposed DOS were computed with the use of the tetrahedron method for integration in the reciprocal k-space. The energy dispersion curves were calculated in the first bcc Brillouin zone along the high-symmetry directions. In the case of the compounds Cu_12_Sb_4_S_12_ and Cu_12_Sb_4_S_13_, their electronic structure was also calculated using the full-potential KKR method, which enables a comparison with the KKR-CPA results obtained within the spherical crystal potential of the muffin-tin form.

## 3. Results and Discussion

### 3.1. Structural Analysis

**Figure 1:**
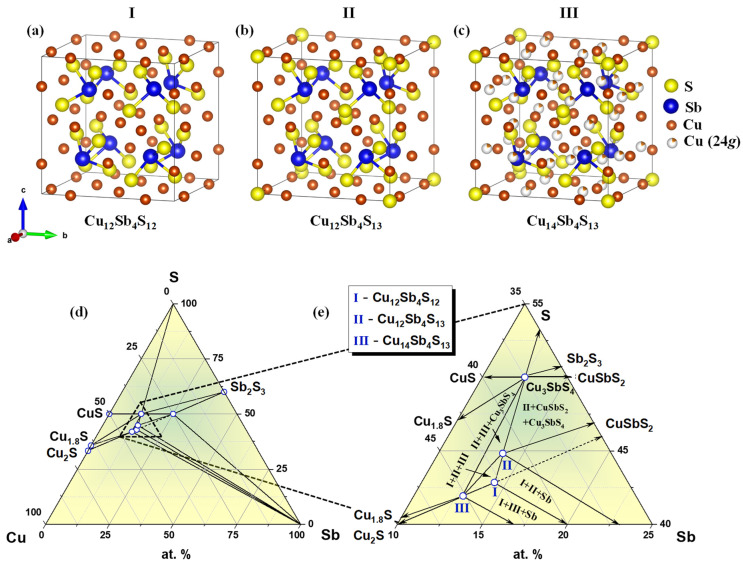
Crystal structures of Cu_12_Sb_4_S_12_ (**a**), Cu_12_Sb_4_S_13_ (**b**), and Cu_14_Sb_4_S_13_ (**c**); Cu-Sb-S phase diagram at 300 K (**d**) with magnification in the vicinity of tetrahedrite phases (**e**).

Investigations of the Cu−Sb−S phase diagram suggest that the tetrahedrite structure could be obtained over a wide compositional range from Cu_12_Sb_4_S_12_ to Cu_14_Sb_4_S_13_ (Figure 1). The well-known Cu_12_Sb_4_S_13_ tetrahedrite mineral crystallizes in a complex cubic structure (space group *I*-43*m* (No. 217)) with a large unit cell of 58 atoms (Figure 1b). There were two different Wyckoff positions (Table 1), where the Cu(1) atoms (12*d*) were tetrahedrally coordinated by four sulfur atoms, whereas the Cu(2) atoms (12*e*) had a triangular environment formed by three sulfur atoms in nearly coplanar coordination. The Sb atoms occupied tetrahedral 8c sites but were bonded to only three S atoms, leaving 5*s* lone pair electrons in the structure [47]. The crystal structure of Cu_12_Sb_4_S_13_ contained unoccupied sites, which could be partially occupied by the additional Cu(3) atoms (24*g*). In such a case, the Cu-rich tetrahedrite Cu_14_Sb_4_S_13_ with 62 atoms per unit cell could be obtained (Figure 1c [48,49]). The Cu(3) atoms, similar to Cu(2), had triangular coordination by three S atoms. Cubic modification of skinnerite, or so-called pseudotetrahedrite, with a chemical composition of Cu_12_Sb_4_S_12_ (or Cu_3_SbS_3_), had the same structural organization as tetrahedrite. However, it had only 56 atoms in the unit cell due to the absence of S(2) atoms in the 2*a* position (Figure 1) [50].

The ternary phase diagram of the Cu-Sb-S system at 300 K (Figure 1d,e) was reconstructed using data presented in Refs. [51] and [52]. Besides, the well-known CuSbS_2_ (chalcostibite), Cu_3_SbS_4_ (famatinite), and Cu_12_Sb_4_S_13_ (tetrahedrite, phase II) ternary compounds, the Cu_12_Sb_4_S_12_ (phase I) and Cu_14_Sb_4_S_13_ (phase III) are additionally presented in the form of separate phases. The Cu_14_Sb_4_S_13_ (phase III) forms tie lines with elemental antimony and with the Cu_12_Sb_4_S_13_ (phase II) (Figure 1d,e).

**Figure 2:**
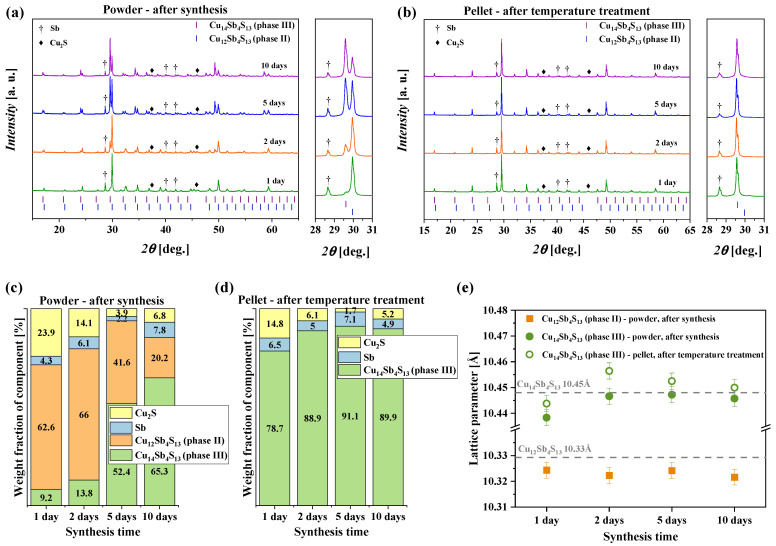
X-ray diffraction patterns of powders after synthesis (**a**) and pellets after temperature treatment (**b**); phase content after synthesis (**c**) and after temperature treatment (**d**); lattice parameters of tetrahedrite products (**e**).

The investigated samples with nominal compositions of Cu_14_Sb_4_S_13_ were characterized by powder X-ray diffraction. The powder XRD patterns are presented in Figure 2a,b along with the theoretical Bragg positions of Cu-rich (phase III-Cu_14_Sb_4_S_13_) and Cu-poor (phase II-Cu_12_Sb_4_S_13_) tetrahedrite. The performed phase analysis indicates that the main phase after 1-day synthesis was Cu-poor tetrahedrite (phase II) with a small amount of Cu-rich tetrahedrite (phase III) and by-products in the form of Cu_2_S and Sb (Figure 2a,c). The increase of the synthesis duration caused a content decrease of the Cu-poor tetrahedrite (phase II) and by-products. As a result, the Cu-rich tetrahedrite (phase III) became the dominant phase (Figure 2a,c). Intriguingly, after PECS treatment and annealing of prepared pellets, all samples showed presence of Cu-rich tetrahedrite (phase III) with a minor amount of Cu_2_S and Sb by-products (Figure 2b shows good agreement with the literature data (Figure 2e).

### 3.2. Microstructural Analysis

**Figure 3:**
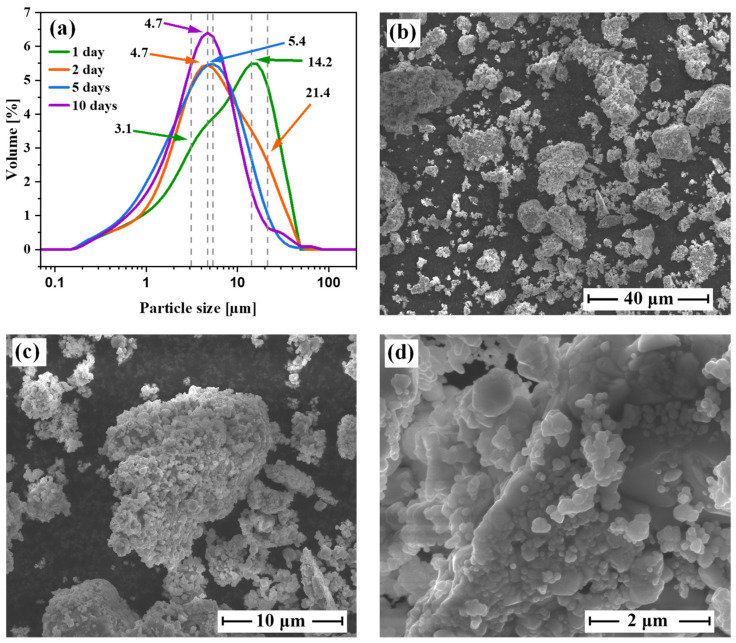
Dynamic light scattering analysis of the investigated powders (**a**), microstructural analysis of the representative Cu-rich tetrahedrite powders after five days synthesis (**b**–**d**).

Figure 3 shows the results of the dynamic light scattering (DLS) analysis and scanning electronic microscopy (SEM) images of the Cu-rich tetrahedrite powders. The DLS data demonstrate that grains after each synthesis ranged from 0.17 µm to 70 µm (Figure 3a). The performed analysis also showed that for the powders obtained after 1 and 2 days, the particle size distributions had two overlapping peaks (Figure 3a). In the case of 1-day synthesis, the number of larger aggregates predominated with the modal value *d* of 14.2 µm. However, their number decreased along with the increase of synthesis time. After 5 and 10 days of synthesis, we observed unimodal particle size distributions with modes *d*_1_ and *d*_2_ of 5.4 and 4.7 µm, respectively. Those results may be related to a larger amount of the substrates present in 1–2 days syntheses, which had grain sizes of ca. 10–20 µm. On the other hand, the products were characterized by smaller grains of 3–6 µm. Therefore, the 5- and 10-day syntheses possess unimodal grain size distributions with smaller grains.

The SEM images, performed for Cu-rich tetrahedrite powder after five days of synthesis, roughly confirmed the DLS data (Figure 3b–d). The sizes of grains observed in SEM varied from tens of micrometers to hundreds of nanometers, which agrees with the results shown in Figure 3a. Moreover, as can be indicated in Figure 3d, the estimated DLS particles probably correspond to the grain aggregates consisting of even smaller nano-scale grains.

### 3.3. Electronic Structure Calculations

**Figure 4:**
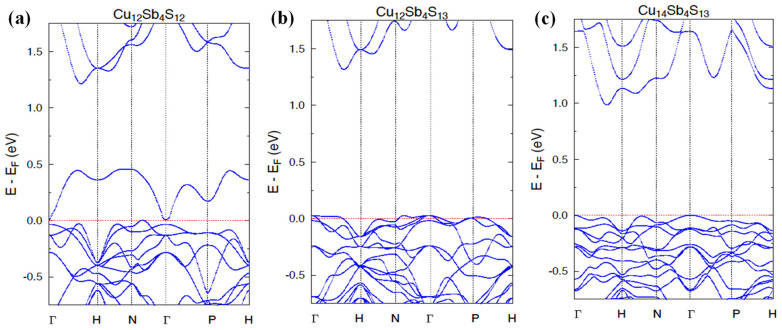
Electronic dispersion curves along high-symmetry directions in the tetrahedrites Cu_12_Sb_4_S_12_ (**a**), Cu_12_Sb_4_S_13_ (**b**), and Cu_14_Sb_4_S_13_ (**c**), calculated by the KKR and KKR-CPA methods. The Fermi level was set to zero.

Figure 4 shows the dispersion curves for Cu_12_Sb_4_S_12_ and Cu_12_Sb_4_S_13_ compounds. Let us remind that the unit cell of the latter contains the additional S atoms located in position 2*a* (0, 0, 0). The dispersion relations E(**k**) of Cu_12_Sb_4_S_13_ are characterized by weakly dispersive valence bands along the Gamma–H, N–H, and N–P directions and by an indirect energy gap of ~1.4 eV, as already reported in previous theoretical studies [35]. The Fermi level lies inside the valence bands (leaving two holes to reach the energy gap), suggesting that the *p*-type metallic character of this compound and the semiconducting properties can be expected when adding two extra electrons to the system (e.g., substituting Sb with Te in Cu_12_Sb_2_Te_2_S_13_ [35]). Unlike in Cu_12_Sb_4_S_12_, the Fermi level was found in the pseudogap, with one strongly dispersive band developed just above this pseudogap. Consequently, the large gap separating the valence and conduction bands—the characteristic electronic structure feature of tetrahedrite and tennantite ternary systems—can be recognized above this single band, but is much smaller (~0.8 eV) than in Cu_12_Sb_4_S_13_.

**Figure 5:**
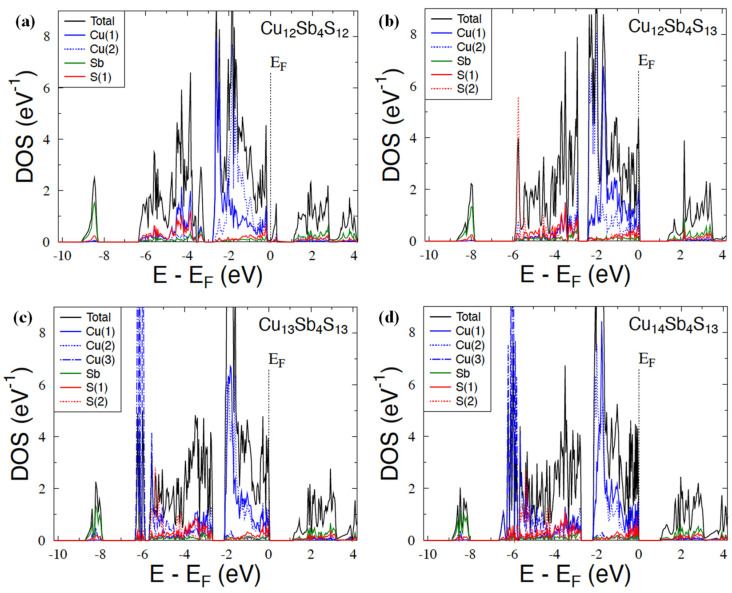
KKR and KKR-CPA total and site-decomposed DOS for Cu_12_Sb_4_S_12_ (**a**) and Cu_12+x_Sb_4_S_12_ with x = 0 (**b**), x = 1 (**c**), and x = 2 (**d**). The Fermi energy (E_F_) was set to zero.

**Figure 6:**
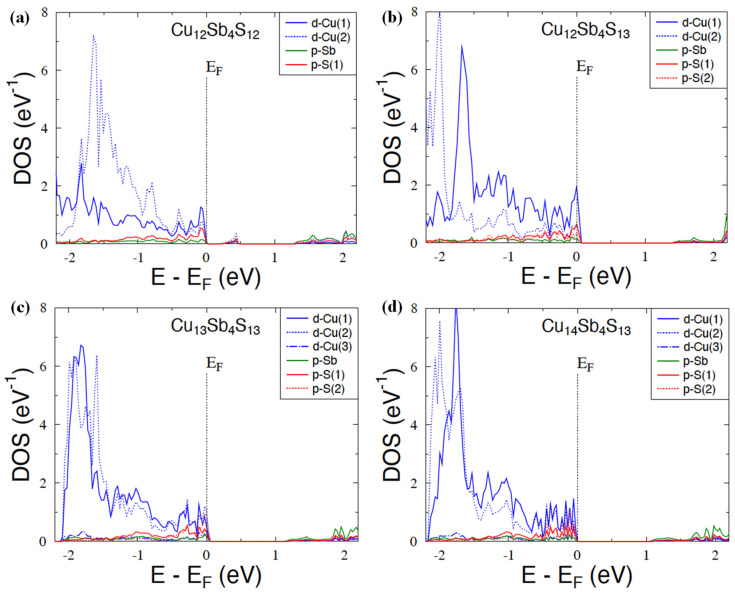
Dominating partial DOS (*d*-states of Cu, *p*-states of Sb, and *p*-states of S) in the vicinity of *E*_F_ in Cu_12_Sb_4_S_12_ (**a**) and Cu_12+x_Sb_4_S_12_ with *x* = 0 (**b**), *x* = 1 (**c**), and *x* = 2 (**d**). The Fermi energy was arbitrarily set to zero.

We suppose that such important modifications of the electronic band structure in the two seemingly close compounds arrive from intense electronic interactions between S(2*a*) and the surrounding Cu atoms. It appears to be responsible for stronger *p-d* hybridization, leading to the formation of a more compact block of valence states with respect to the single band seen in Cu_12_Sb_4_S_12_. This electronic structure behavior can be well seen in Figure 5, presenting the total and site-decomposed DOS in both compounds. Indeed, E_F_ is detected in either the large peak or deep minimum of DOS in Cu_12_Sb_4_S_13_ (Figure 5b) and Cu_12_Sb_4_S_12_ (Figure 5a), respectively. It is worth noting that the interactions between S(2a) and Cu(1) and Cu(2) atoms are quite different since the *d*-states of Cu(1) markedly dominated at the Fermi level in comparison with the *d*-Cu(2) states in Cu_12_Sb_4_S_13_ (Figure 6b). This is probably due to the much shorter S(2)-Cu(2) interatomic distance of ~2.2 A yielding strong bonding, with respect to ~5.7 A observed for the S(2*a*)-Cu(1) one. Conversely, in Cu_12_Sb_4_S_12_ (having 2*a* site vacant), the *d*-state contributions of Cu(1) and Cu(2), forming the large DOS peak just below E_F_, were comparable (see Figure 6a).

As already mentioned in Section 2.4, we intended to study from the KKR-CPA total energy calculations whether the excess of Cu in the Cu_12+x_Sb_4_S_13_ system was related to occupancy of only the third position Cu(3) (24 g Wyckoff site), or if it additionally modifies the copper atom distribution on the Cu(1) and Cu(2) sites. Accordingly, we have considered a few representative models with different occupancies on the Cu sites (Table 2) in three tetrahedrite alloys Cu_12_Sb_4_S_13_, Cu_13_Sb_4_S_13_, and Cu_14_Sb_4_S_13_. In view of the KKR-CPA results, the model where Cu atoms first occupy the Cu(1) and Cu(2) sites, and the remaining atoms fulfill the Cu(3) site, is energetically the most favorable case, whatever the considered composition. The three above-mentioned compositions accounted for the computations, and the models with partial disorder at all sites appear to have markedly higher total energy, ca. 0.2–0.4 eV (per atom), with respect to the lowest energy model containing partial disorder (vacancies) exclusively on the Cu(3) site. The reasons for the preference for such a filling of crystallographic positions of copper can also be notified when inspecting in more detail the DOSs for the three Cu positions. The *d*-Cu(3) states formed a narrow band just below the block of *d-p* valence states constituted by electrons from Cu(1), Cu(2), Sb and S(1), and S(2) atoms, being well seen in Figure 5c,d. This means that it is energetically more advantageous for electrons to occupy the *d*-Cu(3) states compared to the *d*-Cu(1) and *d*-Cu(2) states, which are much higher on the energy scale. The distinctly different position and shape of DOS (narrow peak) for Cu(3) in relation to the DOS, computed for Cu(1) and Cu(2), also implies the overall evolution of the electronic structure of Cu_12+x_Sb_4_S_13_ with an increase in x concentration.

The weak overlap of the DOS peak of Cu(3), with those of Cu(3) and Cu(1), caused the highest-lying valence states (just below the energy gap) to not be markedly affected with the x increase. It was found that each extra Cu atom occupying the 24*g* sites actually delivered only one electron to the *p-d* block of valence states, shifting E_F_ into the gap edge. The consequence of such the more-or-less rigid band behavior was the appearance of a semiconducting state for the composition Cu_14_Sb_4_S_13_ (x = 2) when the Fermi level fell into the energy gap.

### 3.4. Electrical and Thermal Transport Properties

Transport properties for the investigated Cu-rich tetrahedrites at *T* = 300 K are shown in Table 3. All samples measured in this work showed very high values of the Seebeck coefficients *S*, in the range of 420–440 μVK^−1^. The Cu-poor tetrahedrites, taken for comparison from the literature [56,57], showed much lower values of the Seebeck coefficient (Table 3). This is mainly connected with the ca. two orders of magnitude higher carrier concentration for Cu-poor tetrahedrite (~10^20^ cm^−3^) compared with the Cu-rich one (~10^18^ cm^−3^). The Cu-poor Cu_12_Sb_4_S_13_ tetrahedrite is reported to be a *p*-type metal with two holes per formula unit [58]. In the case of the Cu-rich Cu_14_Sb_4_S_13_ tetrahedrite, two additional Cu atoms per formula unit would largely compensate the holes and materials, preferably exhibiting semiconducting behavior, as shown by our DFT calculations. The chemical potential for the Cu-poor tetrahedrite phase was located deeply in the valence band. However, in the case of the Cu-rich phase, the chemical potential was approaching the bandgap, which led to the semiconducting behavior of electrical transport and high Seebeck coefficients.

On the other hand, the carrier mobility of the investigated samples was rather low (6–9 cm^2^V^−1^s^−1^). Such a strongly disturbed carrier transport could be related to the mobile Cu ions in tetrahedrites, as is proposed in refs. [48,59]. Because the Cu(1) site neighbors the partially filled Cu(3) sites, the migration of copper ions should be mainly located between these positions. As the occupancy of the Cu(1) site was reported to be lower for the Cu_14_Sb_4_S_13_, compared with the Cu_12_Sb_4_S_13_, the more intensive copper ion migration is expected in the Cu-rich tetrahedrite phase [37], which could have led to the low carrier mobility recorded in our samples.

Using the Wiedemann-Franz law and measured values of the electrical conductivity σ, we found that the electronic component of the thermal conductivity κ_el_ was less than 5% for all samples and temperatures investigated in this work. Thus, the thermal conductivity κ for the studied Cu-rich tetrahedrites was mainly defined by the lattice thermal conductivity κ_L_. Therefore, for further analysis, we assumed that κ = κ_L_. The lattice thermal conductivity κ_L_ for Cu-rich tetrahedrites at *T* = 300 K was in the range of 0.24–0.32 W m^−1^ K^−1^. Such ultralow κ_L_ values were comparable to those in Cu_12_Sb_4_S_12_ tetrahedrites (0.30 W m^−1^ K^−1^, phase I [57]); however, it was much lower than that of the common Cu_12_Sb_4_S_13_ (0.85 W m^−1^ K^−1^, phase II [56]) tetrahedrite. This phenomenon could also be connected with the liquid-like nature of copper ions, which is more probable in Cu-rich tetrahedrite phases. As is often reported for the superionic argyrodites [24,60], as well as tetrahedrites [48,61,62], the migration of Cu ions can strongly disturb phonon propagation. To verify the TE performance of the investigated samples, the density of the state effective mass m^*^ was calculated using the Kane band model. Details of the calculations can be found in refs. [12,13]. Cu-rich tetrahedrites have the effective mass m^*^ around 0.9 *m*_e_, which is in between Cu_12_Sb_4_S_12_ (phase I) ~0.68 *m*_e_ [57] and Cu_12_Sb_4_S_13_ (phase II) ~1.3 *m*_e_ [56].

**Figure 7:**
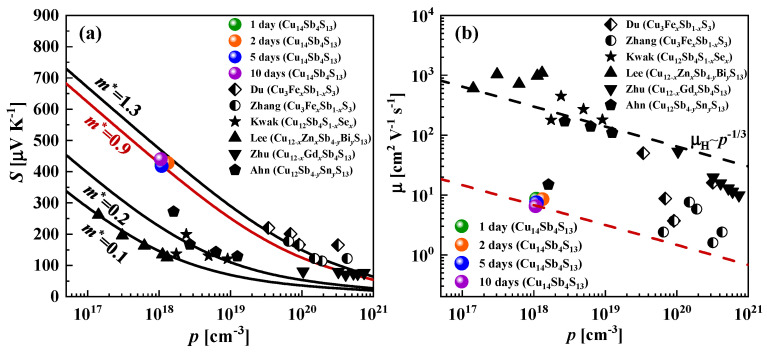
Carrier-dependent Seebeck coefficient (**a**) and mobility (**b**) of the investigated Cu-rich tetrahedrite materials compared with literature data [50,56,57,63,64,65]. Solid lines depict the *S*(*p*) dependences calculated using the Kane band model and considering different effective masses. Dashed lines show the dependence of carrier mobility and carrier concentration as m~*p*^−1/3^, which assumes the case of a constant carrier relaxation time [66,67].

To better understand the transport properties of the investigated tetrahedrite materials, we plotted the Seebeck coefficient and Hall mobility as a function of the carrier concentration (Figure 7). The concentration-dependent Seebeck coefficient at 298 K was calculated using the Kane band model, considering acoustic phonons as the main scattering mechanism (scattering parameter *r* = 0). Within this model, the Seebeck coefficient *S* could be defined using the following expression [3,13]:

(1) $$S=-\frac{k_{B}}{e}\left[\frac{I_{r+1,2}^{1}\left(\eta ,\beta \right)}{I_{r+1,2}^{0}\left(\eta ,\beta \right)}-\eta \right]$$

where *k_B_*, *e*, *r*, and *η* denote the Boltzmann constant, charge of an electron, scattering parameter, and reduced chemical potential, respectively. $I_{n,k}^{m}\left(\eta ,\beta \right)$ are the two-parametric Fermi integrals, which were calculated as follows:

(2) $$I_{n,k}^{m}(\eta ,\beta )=\int_{0}^{\infty }\left(-\frac{df}{dx}\right)\frac{x^{m}{\left(x+\beta x^{2}\right)}^{n}dx}{{\left(1+2\beta x\right)}^{k}}$$

With the obtained values of *η*, carrier concentration *p* could be calculated by the following expression:

(3) $$p=\frac{{\left(2m^{*}{}_{}k_{B}T\right)}^{3/2}}{3\pi^{2}\hbar^{3}}I_{3/2,0}^{0}\left(\eta ,\beta \right)$$

where *N* is band degeneracy and m* is the density of states effective mass. The calculated dependence of the Seebeck coefficient *S*, as a function of carrier concentration *p*, assuming different effective masses, is shown by the solid lines in Figure 7a.

The effective masses of the reported Cu-poor tetrahedrites varied significantly from 0.1 *m*_e_ up to 1.3 *m*_e_; however, the large effective masses were recorded mainly for the tetrahedrites with high carrier concentration (10^19^ cm^−3^–10^21^ cm^−3^). For the case of the investigated Cu-rich tetrahedrites in this work, the relatively high effective mass (m* = 0.9 *m*_e_) is accompanied by the low carrier concentration (*p* ≈ 10^18^ cm^−3^). Such a tendency agreed well with the performed DFT calculations. As discussed above, the mobility μ for the investigated Cu-rich tetrahedrite materials was lower compared with the Cu-poor tetrahedrite materials, which can also be connected with the liquid-like nature of the Cu ions.

**Figure 8:**
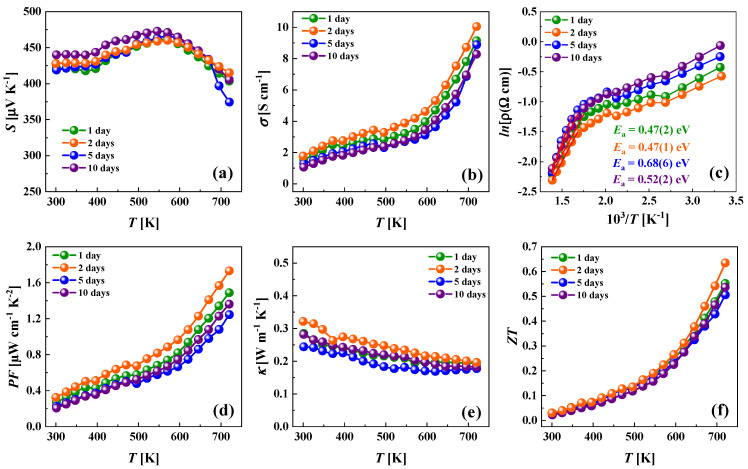
Seebeck coefficient (**a**); electrical conductivity (**b**); Arrhenius plot of electrical resistivity (**c**); power factor (**d**); thermal conductivity (**e**); and *ZT* parameter (**f**) of the investigated Cu-rich tetrahedrite.

The temperature-dependent results of the thermoelectric property measurements of the annealed samples are presented in Figure 8. All investigated Cu-rich tetrahedrites possess a positive Seebeck coefficient *S* over the entire temperature range, indicating that holes are the dominant charge carriers (Figure 8a). Values of the Seebeck coefficient are much higher than reported previously for Cu-rich (80–225 μVK^−1^ [48]) or Cu-poor (60–140 μVK^−1^ [25]) tetrahedrites. The record-high values of the Seebeck coefficient could be attributed to low carrier concentration and relatively high charge carrier effective mass, as discussed above. The Seebeck coefficient was nearly temperature independent in the range of 300–400 K for all samples, and then a characteristic increase of *S* from 400 K up to 575 K was observed. Similar trends of *S* for the Cu-rich tetrahedrite were already reported by Vaqueiro et al. [37,48]. This phenomenon was attributed to the superionic phase transition in Cu-rich tetrahedrites, which cause copper ion migration [48,61]. In the temperature range of 575–725 K, the Seebeck coefficient showed a decreasing tendency, which is typical for intrinsic semiconductors.

The electrical conductivity as a function of temperature is shown in Figure 8b. Overall, the dependence of σ(*T*) resembles the typical semiconductor behavior for all prepared materials. In the temperature range of 300–575 K, a weak increasing tendency of electrical conductivity was observed, which could be attributed to the self-doped region of an intrinsic semiconductor. Such behavior is also indicated on the Arrhenius plot of electrical resistivity (Figure 8c) with activation energies *E*_a_ of 0.1–0.15 eV in this temperature region. Above 575 K, a strong growing tendency of electrical conductivity with temperature could be ascribed to the intrinsic region of the semiconductor with activation energies *E*_a_ of 0.47–0.68 eV (Figure 8c). The obtained values of σ = 1–10 Scm^−1^ for the investigated temperature range were lower than values usually obtained for Cu-poor (400–1000 Scm^−1^ [25]) or Cu-rich (20–200 Scm^−1^ [48]) tetrahedrites for similar temperatures. This could be the result of the low carrier concentration and low carrier mobility, which supports literature statements of Cu-ion liquid-like behavior in Cu-rich tetrahedrites. At 370 K, noticeable discontinuities in a monotonic increase of σ could be attributed to the phase transitions of the Cu_2_S secondary phase [68,69]. However, this effect, as well as a small dip at 480 K, also overlaps with endothermal effects of the main Cu-rich tetrahedrite phase, as was noticed by Vaquiero et al. [48]. 

Power factors (*PF*) for Cu-rich Cu_14_Sb_3_S_13_ tetrahedrite materials are depicted as a function of temperature in Figure 8d. Due to the very high Seebeck coefficient, the *PF* for the best sample achieved 1.7 μWcm^−1^K^−2^, which is a quite promising value among other Cu-based sulfides.

The complex unit cell of Cu-rich tetrahedrite with partial occupancy of Cu sites encourages the migration of Cu ions and leads to the ultralow thermal conductivity over the investigated temperature range. Particularly, the κ shows decreasing tendency from ~0.24–0.32 Wm^−1^K^−1^ at 300 K to 0.18–0.22 Wm^−1^K^−1^ at 725 K. The lowest thermal conductivity κ = 0.17 at 625 K was detected for the material after 5-day synthesis, which is among the lowest values observed in tetrahedrite materials (Figure 8e).

The calculated values of dimensionless thermoelectric figures of merit *ZT* as a function of temperature are shown in Figure 8f. Even if values of the power factor are moderated for the investigated Cu-rich tetrahedrites, high values of the *ZT* parameters were obtained, mainly due to ultralow thermal conductivity. The *ZT* parameters were rising over the investigated temperature range and did not observe a bipolar effect (in the decrease of *ZT*) due to a relatively large bandgap in tetrahedrites (1–1.4 eV [35,70]). The highest value of the thermoelectric figure of merit *ZT* = 0.65 at 725 K was found with the material synthesized at 2 days. Further enhancement of the thermoelectric performance for the investigated Cu-rich tetrahedrites is expected after careful attuning the carrier concentration.

## 4. Conclusions

In this work, we prepared and characterized a Cu-rich Cu_14_Sb_4_S_13_ tetrahedrite compound obtained by a new solvothermal method. The influence of Cu excess in Cu_12+x_Sb_4_S_13_ on the electronic properties was studied using electronic structure calculations by the KKR-CPA method, accounting for the presence of Cu vacancy defects and chemical disorder on three inequivalent Cu sites. The problems of co-existence of two tetrahedrite phases with different stoichiometry and different occupancies of Cu sites, as well as the tendency of tetrahedrite structure to release Cu atoms, were addressed in total energy analyses. The determined impact of increasing Cu content on the character of electronic bands in the vicinity of the Fermi energy was discussed in the context of potential thermoelectric properties.

Cu-rich tetrahedrite phases were prepared using a modified solvothermal method and piperazine, serving both as solvent and reagent. Obtained Cu_14_Sb_4_S_13_ tetrahedrite materials were characterized by very high Seebeck coefficient values (above 400 μVK^−1^), which can be explained by the measured low carrier concentration (~10^18^ cm^−3^) and derived by a DFT *E*_F_ position close to the bandgap edge in Cu-rich tetrahedrite. The temperature-dependent transport properties can be well explained considering the liquid-like nature of the Cu ions in Cu-rich tetrahedrites. Particularly, the low carrier mobility and ultra-low thermal conductivity (*κ* = 0.17–0.32 Wm^−1^K^−1^ at the temperature range of 300–625 K) are good arguments supporting the above statement. Even moderated power factors combined with ultralow thermal conductivity yield an excellent dimensionless thermoelectric figure of merit *ZT* ≈ 0.65 at 723 K for Cu-rich tetrahedrite samples. Further enhancement of power factors through carrier concentration tuning and maintaining ultralow thermal conductivity will lead to an even higher thermoelectric performance of Cu-rich tetrahedrites.

## Figures and Tables

**Table 1:** Crystal structure parameters of Cu_12_Sb_4_S_12_ [53], Cu_12_Sb_4_S_13_ [54], and Cu_14_Sb_4_S_13_ [52,55].

Nominal Composition		Cu_12_Sb_4_S_12_ (Phase I)				Cu_12_Sb_4_S_13_ (Phase II)				Cu_14_Sb_4_S_13_ (Phase III)			
Space group		*I*-43*m* (No. 217)											
*a*/Å		10.240				10.329				10.448			
Unit cell volume/Å^3^		1073.74				1102.08				1140.51			
Z		2				2				2			
Atom	Site	x	y	z	SOF	x	y	z	SOF	x	y	z	SOF
Cu(1)	12d	0.2500	0.5000	0.0000	1	0.2500	0.5000	0.0000	1	0.2500	0.5000	0.0000	1
Cu(2)	12e	0.2500	0.0000	0.0000	1	0.2150	0.0000	0.0000	1	0.2157	0.0000	0.0000	1
Cu(3)	24g	-	-	-	-	-	-	-	-	0.2851	0.2851	0.0102	0.167
Sb	8c	0.2780	0.2780	0.2780	1	0.2682	0.2682	0.2682	1	0.2663	0.2663	0.2663	1
S(1)	24g	0.1250	0.1250	0.3750	1	0.1152	0.1152	0.3609	1	0.1137	0.1137	0.3613	1
S(2)	2a	-	-	-	-	0.0000	0.0000	0.0000	1	0.0000	0.0000	0.0000	1

**Table 2:** Results of the KKR-CPA total energy computations in Cu_12+x_Sb_4_S_13_ models with different occupancies of Cu sites. ΔE = E_min_-E_tot_ denotes the total energy difference between the considered models with various copper atom distributions on the Cu(1), Cu(2), and Cu(3) sites (ΔE is given per atom). E_min_ is the lowest total energy among these models of each composition, suggesting that Cu atoms first occupy the Cu(1) and Cu(2) sites, and the only remaining atoms fulfill the Cu(3) site.

Cu_12_Sb_4_S_13_				Cu_13_Sb_4_S_13_				Cu_14_Sb_4_S_13_			
ΔE (eV)	Cu(1)	Cu(2)	Cu(3)	ΔE (eV)	Cu(1)	Cu(2)	Cu(3)	ΔE (eV)	Cu(1)	Cu(2)	Cu(3)
0	6	6	0	0	6	6	1	0	6	6	2
+0.20	5	6	1	+0.39	5	6	2	+0.29	4	6	3
+0.19	6	5	1	+0.40	6	5	2	+0.32	6	4	3

**Table 3:** The Seebeck coefficient *S*, electrical conductivity σ, lattice thermal conductivity κ_L_, Hall concentration *p*_,_ carrier mobility *µ,* and DOS effective mass m* for investigated specimens at *T* = 300 K.

Sample	*S*, μVK^−1^	*σ*, Scm^−1^	κ_L_, Wm^−1^K^−1^	*p*, cm^−3^	*µ*, cm^2^V^−1^s^−1^	m*/m_e_
1 day (Phase III)	421	1.53	0.28	1.08 × 10^18^	8.7	0.9
2 days (Phase III)	428	1.78	0.32	1.32 × 10^18^	8.5	0.9
5 days (Phase III)	418	1.28	0.24	1.09 × 10^18^	7.4	0.9
10 days (Phase III)	440	1.07	0.28	1.06 × 10^18^	6.5	0.9
Cu_12_Sb_4_S_12_ (Phase I) [57]	166	5.4	0.30	9.1 × 10^19^	3.7	0.68
Cu_12_Sb_4_S_13_ (Phase II) [56]	71	1030	0.85	4.1 × 10^20^	15.7	1.3
